# Effects of age and gender on reference levels of biomarkers comprising the pediatric Renal Activity Index for Lupus Nephritis (p-RAIL)

**DOI:** 10.1186/s12969-017-0202-0

**Published:** 2017-10-13

**Authors:** Michael R. Bennett, Qing Ma, Jun Ying, Prasad Devarajan, Hermine Brunner

**Affiliations:** 1 Division Nephrology and Hypertension, Cincinnati Children’s Hospital Medical Center, Cincinnati, OH USA; 2 Environmental Health, University of Cincinnati College of Medicine, Cincinnati, OH USA; 3 Rheumatology, Cincinnati Children’s Hospital Medical Center, Cincinnati, OH USA

**Keywords:** Lupus nephritis, Gender, Reference range, NGAL, Kim-1, MCP-1, Adiponectin, Ceruloplasmin, Hemopexin, Children

## Abstract

**Background:**

Systemic Lupus Erythematosus (SLE) is a multisystem autoimmune disease that disproportionately effects women and children of minorities. Renal involvement (lupus nephritis, or LN) occurs in up to 80% of children with SLE and is a major determinant of poor prognosis. We have developed a non-invasive pediatric Renal Activity Index for Lupus (p-RAIL) that consists of laboratory measures that reflect histologic LN activity. These markers are neutrophil gelatinase associated lipocalin (NGAL), kidney injury molecule-1 (KIM-1), monocyte chemotactic protein (MCP-1), adiponectin (APN), ceruloplasmin (CP) and hemopexin (HPX). A major gap in the knowledge base and a barrier to clinical utility is how these markers behave in healthy children. We set out to establish a reference range for the p-RAIL markers in a population of healthy children, and to determine if levels of these markers fluctuate with age or gender.

**Methods:**

Urine was collected from 368 healthy children presenting to Cincinnati Children’s primary care clinic for well child visits and assayed for NGAL, KIM-1, MCP-1, APN, CP and HPX using commercially available kits or assay materials.

**Results:**

Specimens were grouped by age (0–5 years (*n* = 94); 5–10 (*n* = 89); 10–15 (*n* = 93); 15–20 (*n* = 91)) and gender (M = 184, F = 184). For age and gender comparisons, values were log transformed prior to analysis. The medians (minimums, maximums) of each marker in the combined population were as follows: NGAL 6.65 (0.004, 391.52) ng/ml, KIM-1416.84 (6.22, 2512.43) pg/ml, MCP-1209.36 (9.49, 2237.06) pg/ml, APN 8.05 (0.07, 124.50) ng/ml, CP 465.15 (8.02, 7827.00) ng/ml, HPX 588.70 (6.85, 17,658.40)ng/ml. All p-RAIL biomarkers but adiponectin had weak but significant positive correlations with age, with NGAL being the strongest (*r* = 0.33, *p* < 0.001). For gender comparisons, NGAL, CP and HPX were elevated in females vs males (86%, *p* < 0.0001; 3%, *p* = 0.007, and 5%, *p* = 0.0005 elevation of the log transformed mean, respectively).

**Conclusions:**

We have established a reference range for the p-RAIL biomarkers and have highlighted age and gender differences. This information is essential for rational interpretation of studies and clinical trials utilizing the p-RAIL algorithm.

## Background

Systemic lupus erythematosus (SLE) is an inflammatory autoimmune disease with multi-organ involvement. Renal involvement in the form of lupus nephritis (LN) is one of the main determining factors in poor prognosis [1]. Childhood-onset SLE (cSLE) [2] typically presents with more severe multi-system disease, including the development of LN in up to 80% of patients, a 10–30% higher proportion than in adult SLE [3–6].

The gold standard of diagnosis of renal involvement in SLE remains histological findings on kidney biopsy [7]. The 3 main patterns of injury used in histological diagnosis and characterization include, mesangial, endothelial and epithelial. These findings are the basis for categorization in the International Society of Nephrology/Renal Pathology Society (INS/RPS) classification system [8]. Due to invasiveness and cost considerations, it is not often practical to perform serial biopsies to track changes in LN such as worsening disease or response to treatment [9]. As a result, conventional laboratory measures are employed, such as changes in proteinuria, complement levels, and anti-ds DNA levels. These measures are not responsive enough to changes, and cannot differentiate activity from damage. Therefore, they are not well suited to direct treatment [10–12].

In response to the shortcomings of conventional measures, we and others have described novel urinary biomarkers (UBMs) that can assist with LN diagnosis, anticipation of flares, [13–16] and correlate with specific histologic changes associated with LN [17]. We recently developed a Renal Activity Index for Lupus Nephritis (RAIL), which was able to predict LN National Institutes of Health Activity Index (NIH-AI) scores with >92% accuracy and tubulointerstitial activity index (TIAI) scores with >80% accuracy [18, 19]. The biomarkers comprising the RAIL include neutrophil-gelatinase associated lipocalin (NGAL), kidney injury molecule 1 (KIM-1), monocyte chemotactic protein 1 (MCP-1), ceruloplasmin (CP), adiponectin (ADP) and hemopexin (HPX). In order to provide greater clinical utility of this panel of markers, we must first understand how these markers behave in healthy individuals, and whether their concentrations change with age or gender. We have previously published reference range and age/gender data for NGAL and KIM-1 [20]. In this study, we set out to establish normative values for MCP-1, CP, ADP and HPX in addition to NGAL and KIM-1 and to determine the effects of age and gender on these normal concentrations.

## Methods

### Patients

This study was approved by the Cincinnati Children’s Hospital Medical Center Internal Review Board and was carried out in accordance with the Declaration of Helsinki. Similar to our previous study [20], samples included were from the Cincinnati Genomic Control Cohort (CGCC). Inclusion criteria were as follows: between 3 years and 18 years of age (prior to 18th birthday) at the time of enrollment, willingness of family to participate and give consent to participate in this project, willingness for participants aged 11 years of age and older to provide assent to participate in this project, ability to complete the introductory medical history, willingness to be contacted annually for future medical history updates, willingness to consent to long-term storage and future analysis of DNA. Subjects were excluded if they met any of the following criteria: presence of known genetic diseases or severe chronic medical conditions, such as chromosomal abnormality, first degree relative participating in the project, unwillingness to complete family and personal health history or allow storage or genetic testing of samples, and adopted, without full contact with biological parent(s) to be able to obtain family history information. Specific exclusion criteria for the subset of patients used in our study was a history of kidney injury or disease, including, but not limited to IGA Nephropathy, kidney stones, abnormal bladder, urinary reflux and ureteral re-implantation.

Recruits were obtained through a marketing plan developed to ensure community based participation, designed with the help of the Clinical Trials Office. Census tract monitoring was used to ensure both the diversity of cohort as well as the representativeness (an equal number of males and females, and approximately 85% white non-Hispanic, 12% African-American, and 3% Asian, Hispanic and other minorities, which represents the population distribution of the 7 counties of Northern Kentucky and Ohio that comprise Greater Cincinnati).

Potential subjects recruited from the community were screened by telephone to ensure eligibility and scheduled for an approximately 2–4 h visit. At this visit, a questionnaire was administered by the clinical research coordinator, a brief physical exam from a licensed physician was performed and samples (blood, urine, hair) were collected. Random urine samples were collected in 4 oz sterile specimen containers. The specimen was then given to the lab 15–60 min after collection, where it was centrifuged briefly to settle particulate matter and aliquoted prior to storage at -80^o^ C. Samples were collected from 2007 to 2010 and stored until measurement in 2015. All measurements were performed in one batch in a period of one week.

### Biomarker measurements

The urine NGAL ELISA was performed using a commercially available assay (NGAL ELISA Kit 036; Bioporto, Grusbakken, Denmark) that specifically detects human NGAL [21]. The intra-assay coefficient of variation (CV’s) was 2.1% and inter-assay variation was 9.1%. The urine KIM-1 ELISA was constructed using commercially available reagents (Duoset DY1750, R&D Systems, Minneapolis, MN) as described previously [22]. Intra and inter-assay CV’s for KIM-1 was 2% and 7.8%, respectively. Adiponectin [CV inter/intra: 4.0%/9.9%] was measured using a commercially available ELISA Kit (R&D Systems, Minneapolis, MN); ceruloplasmin [CV inter/intra: 4.1% /7.1%] and hemopexin [CV inter/intra: 4.8%/7.3%] were also measured by commercially available ELISA kits (Assaypro, St.Charles, MO). MCP-1 was measured by ELISA (R&D Systems, Minneapolis, MN). Intra and interassay CVs for MCP-1 were 5.0% and 5.9%, respectively. Urine creatinine measurements were made using an enzymatic assay, and microalbumin (MALB) was measured by immunoturbidimetry, both on a Dimension RXL plus HM Clinical Analyzer (Siemens, Munich, Germany). Coefficients of variability for the creatinine measurements were 2.4% (intra) and 4.2% (total), and 2.9% (intra) and 5.9% (inter) for MALB.

### Statistical analysis

Means and 95% confidence intervals were calculated from the non-transformed biomarker values using Sigmaplot 13.0 (Systat Software, Inc., San Jose, CA). All biomarkers showed right skewed in their empirical distributions and were corrected using log transformation before analysis, same methods as showed in other publications [13, 16]. For each biomarker, its means were compared between male and female using a two sample t-test. The relationship between the biomarker and the age was assessed using a Pearson correlation coefficient. In addition, aone-way ANOVA model was performed to test the variations of the biomarker between categorized age groups. For a biomarker that showed associations to both age and gender, we firstly estimated the parameters of intercept and slopes using a linear regression model; and then developed an adjusted biomarker that is invariant to age and gender using the estimates. Considering some of the biomarkers showed a pattern of invert U shape in the initial analysis, we started a model (Model 1) with a Age^2^ in the independent variable to fit the shape. The model is proposed in the following where $\widehat{Y}$ denotes a predicted value of the biomarker Y:

1 $$ \widehat{Y}=a+{b}_1\times Age+{b}_2\times Male+{b}_3\times Age\times Male+{b}_4\times {Age}^2+{b}_5\times {Age}^2\times Male $$

Notice Model1 can also be illustrated for male and female respectively in the following:

1.1 $$ \widehat{Y}\mid Female=a+{b}_1\times Age+{b}_4\times {Age}^2 $$

1.2 $$ \widehat{Y}\mid Male=\left(a+{b}_2\right)+\left({b}_1+{b}_3\right)\times Age+\left({b}_4+{b}_5\right)\times {Age}^2 $$

If both *b*_4_ *and b*_5_ are insignificant, we conclude the biomarker is more likely linearly related to Age and the Model1 will be replaced by Model2 in the following:

2 $$ \widehat{Y}=a+{b}_1\times Age+{b}_2\times Male+{b}_3\times Age\times Male $$

Again similarly, the Model 2 can be illustrated for male and female individually in the following:

2.1 $$ \widehat{Y}\mid Female=a+{b}_1\times Age $$

2.2 $$ \widehat{Y}\mid Male=\left(a+{b}_2\right)+\left({b}_1+{b}_3\right)\times Age $$

The Model2 can be further reduced to Model3 should both *b*_1_ *and b*_3_ are insignificant in the estimation.

3 $$ \widehat{Y}=a+{b}_1\times Age $$

The age and gender adjusted biomarker Y* is calculated in the following:

$$ {Y}^{\ast }=Y-\widehat{Y} $$

The validation of the adjusted biomarker was performed after randomly stratifying the entire data into two subsets of training data (75% of the entire data) and testing data (25% of the entire data). Models (1) and (2) were repeated in the training data to estimate the intercepts and slopes. Then the estimates were used in the testing data to develop the adjusted biomarkers. The adjusted biomarkers were tested of the associations to age and gender using the correlation coefficients, ANOVA models, and t-tests respectively.

All statistical tests were performed using SAS 9.4 software (SAS, Cary, NC). Two-sided *p*-values <0.05 were considered statistically significant.

## Results

Urine was collected from 368 children from the Cincinnati Genomic Control Cohort and assayed for NGAL, KIM-1, MCP-1, APN, CP and HPX. Patient demographics can be seen in Table 1. Specimens were grouped by age (0–5 years (*n* = 94); 5–10 (*n* = 89); 10–15 (*n* = 93); 15–20 (*n* = 91)) and sex (M = 184, F = 184). For age and gender comparisons, values were log transformed prior to analysis. The medians (minimums, maximums) of each marker in the combined population were as follows: NGAL 6.65 (0.004, 391.52) ng/ml, KIM-1416.84 (6.22, 2512.43) pg/ml, MCP-1209.36 (9.49, 2237.06) pg/ml, APN 8.05 (0.07, 124.50) ng/ml, CP 465.15 (8.02, 7827.00) ng/ml, HPX 588.70 (6.85, 17,658.40)ng/ml.

**Table 1:** Patient demographics

	3 - < 5 years	5 - < 10 years	10 - < 15 years	15 - < 18 years
n = Males/Females	49/45	45/44	45/48	45/46
Age (mean ± SE)	4.04 ± .06	7.4 ± .15	12.4 ± .15	16.3 ± .1
Height (cm)	103.6 ± .65	124.1 ± 1.4	155.4 ± 1.1	170.4 ± .95
Height z score	0.46 ± .97	0.19 ± 1.1	0.37 ± 0.98	0.36 ± 0.96
Weight (kg)	17.7 ± .35	26.9 ± .82	50.5 ± 1.4	67.1 ± 1.7
Weight z score	0.54 ± 1.1	0.36 ± .98	0.58 ± .96	0.57 ± .99
BMI	16.4 ± .2	18 ± 1.1	20.7 ± .42	23.1 ± .55
BMI z score	0.42 ± 1.2	0.40 ± .94	0.55 ± .87	0.39 ± 1.0
Race %				
● White	86.2	86.5	80.6	82.6
● Black	12.7	13.5	17.2	15.2
● Hispanic	1.1	0	2.2	2.2

z-scores for height weight and BMI were calculated using published CDC growth charts. [42]

In order to determine if there were gender differences between the RAIL biomarkers, raw values were log transformed (natural log) and subjected to a 2-way Student’s t-test. Results can be seen in Table 2. As reported previously [20], NGAL was significantly higher in females than in males (2.52 vs 1.3, *p* = 0.0001). HPX and CP were also higher in females than males (6.58 vs 6.26, *p* = 0.0005; 5.98 vs. 6.16, *p* = 0.007, respectively). These results indicate a need to adjust these markers based on gender.

**Table 2:** Biomarker vs. sex

Variable	Male				Female				
	N	Mean	SD	Upper limit^a^	N	Mean	SD	Upper limit^a^	*p*-value
KIM-1 (pg/ml)	183	5.90	1.00	7.90	183	5.87	0.95	7.77	0.801
NGAL (ng/ml)	184	1.35	1.21	3.77	183	2.52	1.20	4.92	0.0001
MCP-1 (pg/ml)	182	5.28	0.98	7.24	178	5.14	0.97	7.08	0.162
HPX (ng/ml)	184	6.26	0.91	8.08	182	6.58	0.86	8.30	0.0005
CP (ng/ml)	184	5.98	0.70	7.38	182	6.16	0.56	7.28	0.007
ADP (ng/ml)	132	1.85	1.44	4.73	163	1.92	0.84	3.60	0.579
Creatinine (mg/dl)	184	4.70	0.78	6.34	183	4.60	0.71	6.02	0.196
MALB (mg/l)	184	2.42	0.99	4.40	182	2.57	1.03	4.63	0.174

^a^Upper limit of normal = mean + 2 SD

- All values are natural log

To analyze the effects of age on biomarker levels, we subjected the natural log of the means in each age grouping to an ANOVA model and F-test of variance. Results can be seen in Table 3. To summarize, all biomarkers except HPX had a significant association with age, though not always in a predictable direction. Only NGAL, KIM-1 and MCP-1 steadily increased with each age group. While this indicates that age needs to be taken into account when adjusting the RAIL biomarkers clinically, individual ages may need to be taken into account as opposed to simple groupings. To investigate further, Pearson’s tests of correlation was performed between the natural log of biomarker values and the real age (continuous variable) of the subjects (Table 4). NGAL had the strongest positive correlation with age (*r* = 0.33; *p* < 0.0001). All other markers had weak positive correlations with age (*r* = 0.12–0.13), except LFABP and Adiponectin, which both had weak negative correlations with age (*r* = −0.10, *p* = 0.05; *r* = −0.12, *p* = 0.04, respectively).

**Table 3:** Biomarker vs. age group

Variable	Age = 3 - < 5 years			Age = 5 - < 10 years			Age = 10 - < 15 years			Age = 15 - < 18 years			*p*-value of F
	N	Mean	SD	N	Mean	SD	N	Mean	SD	N	Mean	SD	
KIM-1 (pg/ml)	92	5.68	1.01	90	5.78	0.97	92	5.94	0.91	92	6.15	0.97	0.008
NGAL (ng/ml)	93	1.42	1.47	90	1.63	1.30	92	2.08	1.17	92	2.59	1.08	0.0001
MCP-1 (pg/ml)	88	5.00	0.92	89	5.11	0.97	92	5.27	0.98	91	5.45	1.00	0.012
HPX (ng/ml)	92	6.23	0.75	90	6.40	0.65	92	6.56	0.85	92	6.48	1.22	0.082
CP (ng/ml)	92	5.77	0.76	90	6.17	0.47	92	6.20	0.61	92	6.14	0.59	0.0001
ADP (ng/ml)	77	1.95	0.98	71	2.10	0.94	82	1.96	0.98	65	1.50	1.58	0.014
Creatinine (mg/dl)	93	4.19	0.77	90	4.48	0.64	92	4.86	0.59	92	5.07	0.66	0.0001
MALB (mg/l)	92	2.20	0.73	90	2.32	0.72	92	2.72	1.09	92	2.73	1.28	0.0001

-All values are natural log

**Table 4:** Biomarker correlation with real age (continuous variable)

Variable	Pearson’s r	*p*-value
KIM-1 (pg/ml)	0.18	0.0004
NGAL (ng/ml)	0.33	0.0001
MCP-1 (pg/ml)	0.19	0.0003
HPX (ng/ml)	0.12	0.018
CP (ng/ml)	0.19	0.0003
ADP (ng/ml)	−0.12	0.0420
Creatinine (mg/dl)	0.47	0.0001
MALB (mg/l)	0.23	0.0001

-Values calculated fromthe natural log of the biomarkers and real age

Due to statistically significant associations between the RAIL biomarkers and both gender and age, we developed a method for adjusting the levels for males and females as a function of real age (continuous variable). These parameters can be seen in Table 5. For biomarkers of KIM-1, NGAL, MCP-1, HPX, and MALB, their slopes of Age^2^ (or Age x Age) were not significant in either gender. Hence Model 2 fit better for these markers. For the rest of biomarkers of CP, ADP and Creatinine, their slopes of Age^2^ were significant in boys and hence Model1 was preferred for these biomarkers. To illustrate how we calculate an adjusted biomarker, we use KIM-1 as an example for a boy, aged 10 years. Table 5 shows Model 2 is preferred in prediction. The predicted KIM-1 level for this boy will be 5.42 + (0.05 × 10) = 5.97. To return this natural log to a raw value: exp. (5.97) = 391.5 pg/ml. This formula would differ with the same KIM-1 level if the patient were female. With a female patient: 5.42 + (0.03 × 10) = 5.72. To return the natural log to a raw value: exp. (5.72) = 304.9 pg/ml.

**Table 5:** Parameters used for adjusting biomarkers

BMK	Parameter	Male		Female	
		Estimate	StdErr	Estimate	StdErr
KIM-1 (pg/ml)	Intercept	5.42	0.16	5.61	0.16
	Slope	0.05	0.01	0.03	0.01
NGAL (ng/ml)	Intercept	0.17	0.19	1.91	0.19
	Slope	0.12	0.02	0.06	0.02
MCP-1 (pg/ml)	Intercept	4.77	0.17	4.87	0.17
	Slope	0.05	0.01	0.03	0.01
HPX (ng/ml)	Intercept	6.03	0.15	6.36	0.15
	Slope	0.02	0.01	0.02	0.01
CP (ng/ml)	Intercept	5.18	0.10	6.47	0.10
	Slope	0.08	0.01	−0.03	0.01
ADP (ng/ml)	Intercept	2.31	0.23	2.07	0.20
	Slope	−0.05	0.02	−0.01	0.02
Creatinine (mg/dl)	Intercept	3.78	0.11	4.05	0.11
	Slope	0.09	0.01	0.05	0.01
MALB (mg/l)	Intercept	1.78	0.17	2.26	0.17
	Slope	0.06	0.02	0.03	0.02

-Values are natural log

## Discussion

We set out in this manuscript to describe the normative values of the novel urinary biomarkers we established as a pediatric Renal Activity Index for Lupus Nephritis (pRAIL) [18, 19]. We also wanted to determine whether gender and age affected the normative values of these markers. While we and others have reported on reference ranges for urine NGAL and KIM-1 [20, 22–25], this is to our knowledge, the first study to establish urinary reference ranges for MCP-1, ADP, CP and HPX. The need for reference values is important for establishing this panel, not only in children, but adults as well [26].

Most studies investigating biomarkers in lupus nephritis and other chronic conditions use disease controls, such as juvenile idiopathic arthritis (JIA) or SLE patients without renal involvement [13, 27, 28]. Normal behavior of proteins in the healthy population is an important metric in establishing the clinical utility of laboratory tests. Just as proteins will present variability between relatively similar individuals, normal groupings by age and gender often exhibit greater variability and must be taken into account when establishing clinical diagnostic algorithms [20]. In particular, urine proteins have been shown to differ to a greater degree between males and females than in other body fluids, such as cerebrospinal fluid [29].

In this study, we took advantage of the availability of a large cohort of healthy pediatric patients, namely the Cincinnati Genomic Control Cohort. The goal of the development of the cohort was to obtain a population representative sample which could be utilized as controls for a diverse set of projects. Our results showed significant associations of our biomarkers with both age and gender. In concordance with our earlier studies and with published literature, NGAL was significantly higher in females than in males in both pediatric and adult populations [20, 24, 25, 30, 31]. HPX was also found to be significantly higher in females. This has not, to the best of our knowledge, been previously reported. HPX is the primary binder of free heme in the blood. Adult females naturally have lower hemoglobin and associated blood levels of heme due to menstruation, which results in lower HPX levels. In the kidneys, HPX is produced primarily in the renal cortex in the setting of nephrotoxic insults, and acts to protect the tubules from free heme radicals [19, 32]. The reason urine HPX would be elevated in healthy pediatric females would be speculation at this time.

Urine levels of CP were also found to be higher in females. It has been long known that serum levels of CP, a carrier of copper in the blood, are higher in healthy adult females than males and has also been found to increase in older women [33]. While we found an association with age and ceruloplasmin in the pediatric population, it was more of an inverted U shaped association, increasing from age 3 - < 5 up to 10 - < 15, but then decreasing in our oldest grouping, 15 - < 18. In urine, CP increased in response to infections, acting as a molecular source of copper which can inhibit bacterial growth [34]. CP is also a ferroxidase, which can transform ferrous iron, which is toxic to renal tubules, to its nontoxic ferric state. While speculative, CP may be naturally upregulated in females as a host defense due to their greater incidence of UTI compared to males [35].

All of the RAIL proteins showed associations with age, except hemopexin. It is important to note, however, that only NGAL, KIM-1 and MCP-1 had direct positive correlations with age. We have previously shown NGAL and KIM-1 to increase as a function of age [20]. While serum MCP-1 increases with aging in adults, and increases with risk of cardiovascular disease [36], increases in urine MCP-1 in developing children have not been reported to our knowledge. MCP-1 is expressed at high levels in the tubular epithelium with oxidative stress [37] and is predictive of LN flares and LN severity [14, 38]. It would be plausible that there are small increases in subclinical oxidative stress due to environmental exposures in a developing child/adolescent that could potentially lead to increases in tubular expression of MCP-1 in a healthy individual.

“Normalizing” the data for hydration status with urine using creatinine represents a difficult position with a maturing pediatric population. In the growing child, urine creatinine increases as a function of age and maturity [39]. Therefore any “correction” for creatinine applied to our biomarker levels would nullify any increase as a function of age. Indeed, in our population, using a Spearman correlation, urine creatinine demonstrated a significant positive correlation with age (*r* = 0.54, *p* < 0.001). Also, since the pRAIL proteins do not all have a direct positive correlation with age,, creatinine normalization would present additional problems. Since creatinine is dependent upon age in this population, it would not be useful as a normalization tool. It would be important to note that not only does creatinine have an intrinsic relationship with age, it also displays a significant relationship with gender. It is well known that creatinine is higher in males, especially after puberty, than females [40, 41]. As a result, creatinine correction would amplify the difference between males and females in terms of biomarker levels.

The strengths of our study include a large representative healthy pediatric population and established laboratory expertise with the specific methodology used in the study [18, 19]. This study is not without its weaknesses. Our study is from a single center cohort that is representative of the population of a mid-size US city (Cincinnati, Ohio). Demographics from this cohort would differ from other regions and as a result, our results may not be generalizable to the global population. For instance, 85% of our study population classified as White/Non-Hispanic, leaving numbers too small to study race as a variable. Also, while our assays are well established, none of them are standardized assays available on a clinical platform for measurement in urine. As a result, our results may vary from those utilizing different assays. Since the cohort was not originally designed for renal or lupus research, certain pertinent data such as glomerular filtration rate, are not available for these patients. It may be interesting in future studies to investigate other variables, such as hemoglobin levels, which could potentially account for underlying differences between HPX levels in males vs. females.

## Conclusions

Our previous work has demonstrated the utility of the RAIL biomarkers to monitor LN activity in both the pediatric and adult population [18, 19]. The current manuscript has elucidated specific gender and age related associations of the RAIL biomarkers in a population of healthy children. These results have enabled us to develop a method to adjust the levels of the biomarkers for individual patients based on age and sex, to increase the accuracy of our RAIL algorithm. These improvements will increase the clinical utility of the RAIL algorithm and may lead to more effective and personalized treatments for patients with lupus nephritis.

## Abbreviations

ADP: Adiponectin
CP: Ceruloplasmin
HPX: Hemopexin
KIM-1: Kidney injury molecule – 1
LN: Lupus nephritis
MALB: Microalbumin
MCP-1: Monocyte chemotactic protein
NGAL: Neutrophil gelatinase-associated lipocalin
pRAIL: Pediatric renal activity index for lupus nephritis
